# Patients’ Perception in Japan Regarding the Appropriate Use of Antimicrobial Drugs: A Questionnaire Survey

**DOI:** 10.3390/pharmacy11010012

**Published:** 2023-01-09

**Authors:** Hikaru Matsui, Shinya Abe, Taku Obara, Tasuku Sato, Shouko Yoshimachi, Kazuhiko Nomura

**Affiliations:** 1 Tsuruha Holdings Inc., 20-1-21, Kita24-jo higashi, Higashi-ku, Sapporo 065-0024, Hokkaido, Japan; 2 Department of Molecular Epidemiology, Tohoku University Graduate School of Medicine, 2-1, Seiryo-cho, Aoba-ku, Sendai 980-8574, Miyagi, Japan; 3 Department of Pharmaceutical Sciences, Tohoku University Hospital, 1-1, Seiryo-cho, Aoba-ku, Sendai 980-8574, Miyagi, Japan; 4 Department of Clinical Pharmacology and Therapeutics, Tohoku University Graduate School of Pharmaceutical Sciences, 6-3, Aoba, Aramaki, Aoba-ku, Sendai 980-8574, Miyagi, Japan

**Keywords:** antimicrobial drugs, antimicrobial resistance, dispensing pharmacy

## Abstract

This study sought to investigate the actual status of awareness regarding the appropriate use of antimicrobial drugs among patients of a wide age range who visit dispensing pharmacies in order to explore more effective intervention methods for improving awareness of the appropriate use of antimicrobial drugs for patients. A questionnaire survey was conducted with 1301 patients who visited different Tsuruha Holdings-operated pharmacies between 1 September 2018 and 31 October 2018. Using multiple regression analysis, we calculated scores based on the patients’ answers regarding their knowledge of antimicrobial drugs and antimicrobial resistance and examined factors related to these scores. Of the 1185 respondents who successfully completed the survey (mean age ± SD, 52.5 ± 18.2 years), 37.2% were 60 years old or older, 13.2% had never or were not sure whether they had taken antimicrobial drugs, and 73.2% did not understand the problem of antimicrobial resistance. Older age, the non-usage of drugs and self-reports of the “lack” of knowledge of antimicrobial resistance were identified as the group that needs education regarding the appropriate use of antimicrobial drugs. Intervention studies should be conducted to examine the efficacy of interventions based on these factors.

## 1. Introduction

Antimicrobial-resistant bacteria are bacteria that can resist the cytotoxic effects of drugs, and in recent decades, antimicrobial-resistant bacteria that cause infectious diseases have become a problem [1,2]. Antimicrobial-resistant bacteria are linked to longer hospital stays, higher treatment costs, and higher death rates in infected patients compared with sensitive bacteria [3,4]. The necessity to take action against the spread of antimicrobial-resistant bacteria was reported in 2014, and if no action is taken, the number of deaths caused by antimicrobial-resistant bacteria, including bacteremia and pneumonia caused by bacterial infection, is estimated to reach 10 million per year by 2050—exceeding the number of deaths caused by cancer [5]. The occurrence of antimicrobial-resistant bacteria is related to the overuse of antimicrobial drugs and is a problem that involves both medical professionals and patients [6,7,8].

In May 2015, the World Health Organization (WHO) General Assembly adopted a ‘global action plan on antimicrobial resistance’ to tackle the problem of the spread of antimicrobial-resistant bacteria. Member countries were requested to formulate national action plans on antimicrobial resistance within two years; five particular areas of concern were identified: public awareness and education, surveillance and monitoring, the prevention and control of infection, the appropriate use of antimicrobial drugs, and research and development and drug discovery [9]. In Japan, the ‘Action Plan for Antimicrobial Resistance’ was formulated in April 2016, setting goals in seven areas, including the abovementioned five as well as the areas of international cooperation and requiring comprehensive actions [10]. The ‘Action Plan on Antimicrobial Resistance’ includes achievement targets for reducing the use of antimicrobial drugs in medical care and reducing the rate of antimicrobial resistance in major bacterial species [11]. In addition, based on the formulation of this action plan, ‘additional support for the proper use of antimicrobial drugs’ was newly established in the 2018 revision of medical fees, and the activities of support teams for the proper use of antimicrobial drugs in hospitals are now publicly supported [12].

Whereas such support for medical facilities is advancing, a study of public perceptions of antimicrobial drugs in Japan and European countries has shown that the knowledge of antimicrobial drugs and antimicrobial resistance is poor and that antimicrobial drugs are used inappropriately [13]. Therefore, to achieve the goals set out by the ‘Action Plan on Antimicrobial Resistance’ and to reduce the antimicrobial resistance rate, it is important to provide the public with sufficient information and spread knowledge about antimicrobial resistance. The results of an online survey of Japanese people under 70 years confirm that one’s level of education is associated with adequate knowledge of antimicrobial drugs and antimicrobial resistance [14]. However, in Japan, there has been no survey on the knowledge of antimicrobial drugs and antimicrobial resistance involving a wide range of age groups.

The purpose of this study was therefore to investigate the actual status of awareness regarding the appropriate use of antimicrobial drugs among patients who visit dispensing pharmacies, in order to explore more effective intervention methods for improving awareness of the appropriate use of antimicrobial drugs for patients.

## 2. Materials and Methods

A questionnaire survey was conducted with patients who were visiting dispensing pharmacies operated by Tsuruha Holdings. Japanese pharmacies could be divided into three types (pharmacy, drugstore, and dispensing pharmacy). These three are sometimes combined. Patients could receive prescription drugs only at a dispensing pharmacy. A paper questionnaire was employed to survey patients’ perceptions of antimicrobial drugs and antimicrobial resistance. The research was approved by the Human Research Ethics committee of Tsuruha Holdings (Approval No. HD2019008, dated 1 August 2018) before the execution of the study.

From 1 September 2018 to 31 October 2018, we asked 1301 patients visiting Tsuruha Holdings-operated pharmacies in Kanagawa Prefecture, Japan to participate in our questionnaire survey based on their own judgment. The characteristics of the pharmacies that were involved in the questionnaire survey are presented in Table 1. There are two types of dispensing pharmacies in Japan: those that receive prescriptions from specific medical institutions and those that are not from specific medical institutions but are community-based. In the latter, the number of pharmacists is not simply determined based on the number of prescriptions, as they also deliver medicines to homes. The pharmacies covered in this survey fall into the latter, with as few as 10 prescriptions per pharmacist.

The questionnaire used in this study and the responses to each question are shown in Supplementary Materials. We could not test the questionnaire before this survey. Question items on the following were used for analysis: respondents’ age (years), sex (man, woman), and experience with using antimicrobial drugs (Q1. Have you ever taken any antimicrobial drugs?); respondents’ awareness of the efficacy of antimicrobial drugs for colds and influenza (Q4. Antimicrobial drugs are effective for colds. Q5. Antimicrobial drugs are effective for influenza.); respondents’ understanding of antimicrobial drugs (Q8. I understand what antimicrobial drugs are.); respondents’ interruption of antimicrobial drug use on their own judgment (Q9. If I am prescribed antimicrobial drugs at the hospital, I think it is okay to stop taking them when my symptoms improve.); respondents’ use of antimicrobial drugs on their own judgment when they have a cold (Q10. If I had some surplus antimicrobial drugs from a previous prescription, I would take them anyway if I catch a cold again.); whether respondents would like to take antimicrobial drugs when they have a cold (Q11. I would like to take antimicrobial drugs when I have a cold.); respondents’ awareness of the importance of complete taking of antimicrobial drugs as the prescriber intended (Q13. If I am prescribed antimicrobial drugs, I will take them completely.); respondents’ awareness of whether or not they understand antimicrobial resistance (Q14. I understand the problem of drug resistance.); and respondents’ awareness of the issue of antimicrobial resistance (Q15. The problem of antimicrobial resistance does not concern me.). The answers to Q4 to Q17 were five choices: ‘Agree’, ‘Somewhat agree’, ‘Neither’, ‘Somewhat disagree’, and ‘Disagree’ (Supplementary Materials).

### 2.1. Evaluation Items

The answers to question items concerning antimicrobial drugs and antimicrobial resistance were scored as the research evaluation items. Four question items were related to antimicrobial drugs: (1) ‘Antimicrobial drugs are effective for colds’, (2) ‘Antimicrobial drugs are effective for influenza’, (3) ‘If I had some surplus antimicrobial drugs from a previous prescription, I would take them anyway if I catch a cold again’, and (4) ‘I would like to take antimicrobial drugs when I have a cold’. For each of these four question items, one point was given as a correct answer if the participant selected ‘disagree’ or ‘somewhat disagree’, and no points were given if the participant selected ‘neither agree nor disagree’, ‘somewhat agree’, or ‘agree’ as an incorrect answer. The total score obtained for these four items was used as the overall score for the answers to the four question items related to antimicrobial drugs. Similarly, we also scored the answers to question items concerning antimicrobial resistance. We prepared the following three question items regarding antimicrobial resistance: ‘If I am prescribed antimicrobial drugs at the hospital, I think it is okay to stop taking them when my symptoms improve’, ‘If I am prescribed antimicrobial drugs, I will take them completely’, and ‘the problem of antimicrobial resistance does not concern me’. For each of the three question items, one point was given as a correct answer if the participant selected ‘disagree’ or ‘somewhat disagree’, and no points were given if the participant selected ‘neither agree nor disagree’, ‘somewhat agree’, or ‘agree’ as an incorrect answer. The total score for these three question items was used as the overall score for the three answers to the question items related to antimicrobial resistance. However, for the question item ‘If I am prescribed antimicrobial drugs, I will take them completely’, one point was given if the respondent answered ‘agree’ or ‘somewhat agree’, whereas zero points were given if the other options were selected. Additionally, then, a seven-point scale was defined for the total score obtained by adding the scores of the answers to the question items concerning antimicrobial drugs and antimicrobial resistance.

### 2.2. Statistical Analysis

We examined whether age, sex, experience with the use of antimicrobial drugs, and awareness of understanding of antimicrobial drugs and antimicrobial resistance were associated with the total score regarding antimicrobial drugs and antimicrobial resistance. The scores for each stratum were expressed as mean score ± standard deviation (SD) and median score. In addition, multiple regression analysis was conducted for the total score regarding antimicrobial drugs and antimicrobial resistance. As for the awareness of the understanding of antimicrobial drugs and antimicrobial resistance, if participants did not understand, we gave a score of 0. Statistical analyses were conducted using the software SAS^®^ 9.4. The statistical significance level was set at less than 5%.

## 3. Results

### 3.1. Basic Characteristics of the Patients

Of the 1301 patients initially identified for participation, 1185 agreed with this survey and successfully completed the questionnaire as pharmacists were available to assist with responses as needed (91.1% response rate). The basic characteristics of the patients who completed the questionnaire are summarized in Table 2. Of the 1185, approximately 67% were female, and the mean age ± SD was 52.5 ± 18.2 years, covering an age range of 20 to 97 years. More than 80% of the 1185 patients had taken antimicrobial drugs.

### 3.2. Understanding of Antimicrobial Drugs and Antimicrobial Resistance

Table 3 shows the proportion of incorrect answers for each question regarding antimicrobial drugs or antimicrobial resistance stratified by awareness of understanding of antimicrobial drugs. The percentage of participants who incorrectly answered the question item ‘antimicrobial drugs are effective for colds’ was 80.7%. The group that answered that they understood antimicrobial drugs tended to have a higher percentage of incorrect answers to question items regarding antimicrobial drugs.

Table 4 shows the question item scores regarding the respondents’ knowledge of antimicrobial drugs and antimicrobial resistance stratified by age, sex, experience with the use of antimicrobial drugs, and awareness of understanding of antimicrobial drugs and antimicrobial resistance. Sex was not associated with the total score regarding antimicrobial drugs and antimicrobial resistance. Comparing the scores between age groups, the older age group had a lower score. A similar relationship was observed for experience with the use of antimicrobial drugs. The group that had taken antimicrobial drugs tended to have a higher score than the two groups that responded that they had never taken or were not sure if they had ever taken antimicrobial drugs. The total score of the question regarding antimicrobial drugs and antimicrobial resistance was not different between patients who answered ‘I understand what antimicrobial drugs are’ and those who did not. For all of the other evaluation items, the scores were higher for participants who answered that they understood antimicrobial drugs or antimicrobial resistance.

The results of the multiple regression analysis of scores are shown in Table 5. The total score of the question items regarding antimicrobial drugs and antimicrobial resistance was negatively associated with age and the absence of experience with the use of antimicrobial drugs and positively associated with the understanding of antimicrobial resistance. The score of the question item regarding antimicrobial drugs was negatively associated with age and the understanding of antimicrobial drugs, and positively associated with the understanding of antimicrobial resistance. The score of the question about antimicrobial resistance was negatively associated with being male, one’s age, and the absence of experience with the use of antimicrobial drugs, and positively associated with the understanding of antimicrobial drugs and the understanding of antimicrobial resistance.

## 4. Discussion

We found that 13.2% had never or were not sure whether they had taken antimicrobial drugs, and 73.2% did not understand the problem of antimicrobial resistance. Age, experience with the use of antimicrobial drugs, and self-reports of understanding antimicrobial resistance were significantly and independently associated with a correct understanding of antimicrobial drugs and antimicrobial resistance.

### 4.1. Misunderstanding about Antimicrobial Drugs

As colds are commonly caused by viruses, treating them using antimicrobial drugs would be ineffective [15]. However, in our study, the percentage of participants who incorrectly answered the question item ‘antimicrobial drugs are effective for colds’ was more than 80%, regardless of whether they were aware of an understanding of antimicrobial drugs. A previous study on the understanding of antimicrobial drugs among Japanese people found that knowledge of the difference between viral and bacterial infections was poor [13]. Therefore, it is possible that many of the surveyed patients in our study similarly mixed up or did not understand the differences between bacteria and viruses.

The group that answered that they understood antimicrobial drugs tended to have a higher percentage of incorrect answers to question items regarding antimicrobial drugs. This suggests that people who are aware that they understand antimicrobial drugs may not actually have the correct knowledge of antimicrobial drugs. In Japan, the domestic market for antimicrobial products is estimated to be more than one trillion yen [16], and the number of products with ‘antimicrobial’ as an advertising slogan is increasing. Therefore, by hearing the word ‘antimicrobial’ on a daily basis, many people may feel that they have a better understanding of antimicrobial drugs, but may actually have misunderstandings about them.

### 4.2. Factors Related to the Knowledge of Antimicrobial Drugs and Antimicrobial Resistance

Older participants had a higher percentage of those who stated that they understood antimicrobial drugs, but their scores regarding knowledge of antimicrobial drugs were lower than those of younger participants. This suggests that a higher percentage of older participants had misunderstandings about antimicrobial drugs and may be more likely to have an effective experience with interventions. In the multiple regression analysis regarding scores on question items related to understanding antimicrobial drugs and antimicrobial resistance, older age and no experience with the use of antimicrobial drugs were associated with a lower score, and an understanding of the issue of antimicrobial resistance was associated with a higher score. This suggests that intensive instruction for patients who are older, who are taking antimicrobial drugs for the first time, or who are not familiar with the term ‘antimicrobial resistance’ would be effective in ensuring correct knowledge of antimicrobial drugs and antimicrobial resistance.

It was found that whether patients knew about antimicrobial drugs did not relate to their actual knowledge of antimicrobial drugs and antimicrobial resistance. Therefore, when asking patients about their knowledge of antimicrobial drugs, it would be helpful to ask them not only about their awareness of antimicrobial drugs but also about the term ‘antimicrobial resistance’ in order to accurately understand their knowledge of antimicrobial drugs and antimicrobial resistance. In actual clinical practice, it would be effective to ask patients ‘Do you know the term antimicrobial resistance?’ and provide a more detailed explanation to those who answer that they do not know. Therefore, three factors were identified as potential criteria for changing the intensity of instruction: age, experience with the use of antimicrobial drugs, and awareness of the presence or absence of an understanding of antimicrobial resistance. Patients who meet these three criteria are likely to be the most effective targets for intensive instruction to increase public knowledge of antimicrobial drugs and antimicrobial resistance.

Previous studies have shown that patient education level is associated with knowledge of antimicrobial drugs and antimicrobial resistance [17]. A study conducted in Japan also showed that knowledge of antimicrobial drugs and antimicrobial resistance is more influenced by educational level than by age or experience with the use of antimicrobial drugs within one year [14]. Previous surveys were conducted online, and people over 70 years old were not included because they may have difficulty responding online [14]. Therefore, it is likely that different results were obtained due to different explanatory variables and populations between previous surveys and our study. On the other hand, this study surveyed a wide range of age groups, from 20 to 97 years old, and thus obtained differences in perceptions by age that were not revealed in previous studies [14]. In addition, in the case of the internet surveys adopted in previous studies [14], the population may be biased with a high level of internet literacy among the participants, but in this study, a paper-based questionnaire was used among the patients; thus, it is considered to have a higher generalizability. In addition, by focusing on patients visiting dispensing pharmacies, we were able to clarify the current situation regarding the population that should be realistically informed and educated.

In this study, we extracted candidates for criteria to give special emphasis in instruction. Although the impact of the different possible scoring schemes should be examined, at least, the validity of these criteria needs to be tested by conducting intervention studies that focus on whether the three criteria identified actually improve patients’ awareness of antimicrobial drugs and antimicrobial resistance by providing them with guidance. It is also necessary to examine factors not included in this study to further identify targets for intervention and to conduct surveys in wide areas. If the effectiveness of the interventions in pharmacies is recognized, it is expected that pharmacies will be further established as places that promote patient education.

Overall human antimicrobial drug use in Japan in 2019 decreased by 10.9%, oral cephalosporins decreased by 22.7%, fluoroquinolones decreased by 18.1%, macrolides decreased by 20.6%, and intravenous antimicrobial drugs decreased by 12.7% [18]. Although the use and resistance rates of all antimicrobial drugs in Japan, as also reported by other studies [19], are on the way down, the target numbers for 2020 have not been reached; therefore, further efforts are needed.

### 4.3. Research Limitations

In our multiple regression analysis, the value of the adjusted R2 was small, owing to the presence of unmeasured variables. Therefore, we could not sufficiently explain the objective variable. It is thus necessary to enrich the explanatory variables by including data on respondents’ levels of education. Another limitation of this study was that the survey area was limited. It has been reported that regional and geographic diversity exists in the prescription of antimicrobial drugs in the United States, European countries, and Japan [20,21,22,23,24]. Therefore, it is possible that the awareness of antimicrobial drugs may differ by region as well. It is necessary to understand the characteristics of each region and to establish measures for the proper use of antimicrobial drugs. Because our study was conducted before the spread of the coronavirus disease, patients’ perceptions regarding the appropriate use of antimicrobial drugs would be different now. However, we do not have data and could not find information on this topic after the spread of the coronavirus disease. We did not validate and calibrate the questions before administration. There might have been a semantic problem with the way the target patients perceive the word ‘antimicrobial’. Therefore, an additional explanation of the word ‘antimicrobial’ may have been necessary. For the patient, the optimal antimicrobial therapy is to take the drug as prescribed by the physician. However, it is difficult to determine the intrinsic optimal duration of antimicrobial therapy. Therefore, this might make the use of the combined scores difficult.

### 4.4. Research Implications

This study is the first to survey participants of a wide age range covering an age range of 20 to 97 years in Japan regarding their perception of the appropriate use of antimicrobial drugs. Comparing the question item scores regarding the knowledge of antimicrobial drugs and antimicrobial resistance between age groups, those of an older age had a lower score. Therefore, those with misunderstandings about antimicrobial drugs were relatively more likely to be older, which suggests that educational interventions for the elderly are needed.

## 5. Conclusions

Older age, the non-usage of drugs and self-reports of the “lack” of knowledge of antimicrobial resistance were identified as the group that needs education regarding the appropriate use of antimicrobial drugs. Intervention studies should be conducted to examine the efficacy of interventions based on these factors.

## Figures and Tables

**Table 1:** Characteristics of the pharmacies where the survey was conducted.

Dispensing Pharmacy	Mean Number of Prescriptions per Day	Average Number of Full-Time Pharmacists
A	56.3	5.6
B	98.3	9.5
C	68.7	7.7
D	31.0	3.5
E	55.7	5.0
F	77.8	8.1
G	30.6	2.7

**Table 2:** Basic characteristics of the surveyed patients.

	n (%)
Sex	
Male	392 (33.1)
Female	793 (66.9)
Age group	
20–30s	337 (28.4)
40–50s	407 (34.3)
60–70s	349 (29.5)
80s and above	92 (7.8)
Experience with the use of antimicrobial drugs	
Have ever taken antimicrobial drugs	1028 (86.7)
Have not ever taken antimicrobial drugs	78 (6.6)
Do not know if have ever taken antimicrobial drugs	79 (6.7)

**Table 3:** The proportion of incorrect answer for each question regarding antimicrobial drugs or antimicrobial resistance.

		Q8. I Understand What Antimicrobial Drugs Are.	
	Total (n = 1185)	A. Agree or Somewhat Agree (n = 360)	B. Neither, Somewhat Disagree, or Disagree (n = 825)
**Questions regarding antimicrobial drugs**			
Q4. Antimicrobial drugs are effective for colds.			
%	80.7	80.6	80.7
Q5. Antimicrobial drugs are effective for influenza.			
%	71.9	66.9	74.1
Q10. If I had some surplus antimicrobial drugs from a previous prescription, I would take them anyway if I catch a cold again.			
%	30.6	30.3	30.7
Q11. I would like to take antimicrobial drugs when I have a cold.			
%	62.8	65.0	61.8
**Questions regarding antimicrobial resistance**			
Q9. If I am prescribed antimicrobial drugs at the hospital, I think it is okay to stop taking them when my symptoms improve.			
%	33.1	26.7	35.9
Q13. If I am prescribed antimicrobial drugs, I will take them completely.			
%	32.2	24.2	35.6
Q15. The problem of antimicrobial resistance does not concern me.			
%	45.5	33.9	50.6

An incorrect answer means that participant selected ‘neither agree nor disagree’, ‘somewhat agree’, or agree’ as answer for each question.

**Table 4:** Basic characteristics of the surveyed patients.

	n	Total Score Regarding Antimicrobial Drugs and Antimicrobial Resistance Mean Score ± SD, Median Score
**Sex** Male	392	3.42 ± 1.92, 3.00
Female	793	3.44 ± 1.82, 3.00
**Age group** 20–30s	337	3.73 ± 1.83, 4.00
40–50s	407	3.63 ± 1.79, 4.00
60–70s	349	3.16 ± 1.85, 3.00
80s and above	92	2.53 ± 1.78, 2.00
**Experience with the use of antimicrobial drugs**		
Have ever taken antimicrobial drugs	1028	3.56 ± 1.81, 4.00
Have not ever taken antimicrobial drugs	78	2.35 ± 1.95, 2.00
Do not know if have ever taken antimicrobial drugs	79	2.82 ± 1.87, 2.00
**Understanding of antimicrobial drugs**		
Understand	360	3.73 ± 1.85, 4.00
Do not understand	825	3.31 ± 1.84, 3.00
**Understanding of antimicrobial resistance**		
Understand	317	4.41 ± 1.69, 4.00
Do not understand	868	3.08 ± 1.78, 3.00

**Table 5:** Multiple regression analysis on the total score regarding antimicrobial drugs and antimicrobial resistance.

Factors	Partial Regression Coefficient	t-Value	*p*-Value	Partial R^2^
**Sex (Female = 0)**				
Male	−0.071	−0.66	0.508	0.0000
**Age group (20–30s = 0)**				
40–50s	−0.142	−1.12	0.262	0.0059
60–70s	−0.517	−3.89	0.000	0.0046
80s and above	−1.129	−5.45	<0.0001	0.0255
**Experience with the use of antimicrobial drugs (Have ever taken antimicrobial drugs = 0)**				
Have not ever taken antimicrobial drugs	−0.711	−3.4	0.001	0.0125
Do not know if have ever taken antimicrobial drugs	−0.394	−1.93	0.054	0.0070
**Understanding of antimicrobial drugs (Do not understand = 0)**				
Understand	−0.060	−0.51	0.607	0.0098
**Understanding of antimicrobial resistance (Do not understand = 0)**				
Understand	1.336	10.99	<0.0001	0.0870
Total R^2^				0.1524
Adjusted total R^2^				0.1467

## Data Availability

Not applicable.

## Supplementary Materials

The following supporting information can be downloaded at: https://www.mdpi.com/article/10.3390/pharmacy11010012/s1, The questionnaire used in this study.
