# Supplementary material for: Patients’ Perception in Japan Regarding the Appropriate Use of Antimicrobial Drugs: A Questionnaire Survey
Source: Pharmacy (Basel). 2023 Jan 9;11(1):12. doi: 10.3390/pharmacy11010012 (PMC9844386; doi:10.3390/pharmacy11010012)
Supplement: Supplementary file 1 [file pharmacy-11-00012-s001.zip › pharmacy-1981347-supplementary.pdf]

Supplementary Materials. The questionnaire used in this study
(The percentages of selectors for each response are shown in parentheses.)

Questionnaire Survey on the Proper Use of Antimicrobial Agents

Summary
The purpose of this survey is to investigate the awareness of antimicrobial agents.
The information you provide in this survey may be used in internal meetings and presentations at academic conferences, etc., to the extent that individuals cannot be identified. If you agree to this, please check ☐ below.
If you agree to participate in this study, please check the box below to indicate your consent to participate.

☐ I agree to participate in this study.

How to answer
Please answer the following questions as you feel most applicable. If you feel that none of the questions applies to you in particular, please answer the one that is closest to your feeling.
If there is no indication that more than one answer is possible, please put ☑ in one of the boxes.
Please answer in sentences or bullet points in the free answer column.

Age:____years
Sex: ☐Man(33.1%) ☐Woman(66.9%)
Do you have children under 15 years old?: ☐Yes(25.4%) ☐No(74.6%)

1. Have you ever taken any antimicrobial drugs?
☐Yes(86.7%) ☐No(6.6%) ☐I am not sure(6.7%)

2. Have you ever requested a prescription for antimicrobial drugs from your doctor?
(If you have experienced both, please answer one for the most frequent experience.)
☐Yes (answer Question 3)(10.6%) ☐No (skip to Question 4)(89.3%) (No answer, 0.1%)

3. Did the doctor prescribe it at that time?
☐Yes(93.6%) ☐No(4.0%) ☐Do not remember(2.4%)

Please circle the number that you feel most applies to the following question items.

	Agree	Somewhat agree	Neither	Somewhat disagree	Disagree
4. Antimicrobial drugs are effective for colds.	5(23.8%)	4(32.2%)	3(24.7%)	2(5.4%)	1(13.9%)
5. Antimicrobial drugs are effective for influenza.	5(19.9%)	4(23.2%)	3(28.8%)	2(6.5%)	1(21.6%)
6. Antimicrobial drugs are effective for pneumonia.	5(24.3%)	4(28.1%)	3(37.8%)	2(4.7%)	1(5.1%)
7. Antimicrobial drugs are effective for otitis media.	5(22.3%)	4(26.8%)	3(38.7%)	2(5.6%)	1(6.6%)
8. I understand what antimicrobial drugs are.	5(6.3%)	4(24.1%)	3(34.1%)	2(17.1%)	1(18.4%)
9. If I am prescribed antimicrobial drugs at the hospital, I think it is okay to stop taking them when my symptoms improve.	5(10.4%)	4(10.0%)	3(12.7%)	2(15.2%)	1(51.7%)
10. If I had some surplus antimicrobial drugs from a previous prescription, I would take them anyway if I catch a cold again.	5(8.0%)	4(13.7%)	3(8.9%)	2(8.5%)	1(60.9%)
11. I would like to take antimicrobial drugs when I have a cold.	5(12.5%)	4(15.9%)	3(34.4%)	2(9.1%)	1(28.1%)
12. I would like to buy antimicrobial drugs, if they are on sale over the counter.	5(9.6%)	4(13.9%)	3(24.1%)	2(12.9%)	1(39.5%)
13. If I am prescribed antimicrobial drugs, I will take them completely.	5(45.6%)	4(22.3%)	3(13.5%)	2(6.1%)	1(12.5%)
14. I understand the problem of drug resistance.	5(8.9%)	4(17.8%)	3(29.9%)	2(15.9%)	1(27.5%)
15. The problem of antimicrobial resistance does not concern me.	5(4.2%)	4(5.2%)	3(36.1%)	2(15.9%)	1(38.6%)
16. I would like to ask a doctor about	5(15.3%)	4(23.0%)	3(41.8%)	2(7.8%)	1(12.1%)

antimicrobial drugs.					
17. I would like to ask a pharmacist about antimicrobial drugs.	5(16.5%)	4(24.0%)	3(41.5%)	2(6.8%)	1(11.2%)

18. What is your positive image about antimicrobial drugs (free text)?

19. What is your negative image about antimicrobial drugs (free text)?

Thank you very much for your responses.
